# Resolution of Autoimmune Oophoritis after Thymectomy in a Myasthenia Gravis Patient

**DOI:** 10.4274/jcrpe.378

**Published:** 2011-12-06

**Authors:** Esra Deniz Papatya Çakır, Özlem Özdemir, Erdal Eren, Halil Sağlam, Mehmet Okan, Ömer Faruk Tarım

**Affiliations:** 1 Uludağ University School of Medicine, Pediatric Endocrinology, Bursa, Turkey; 2 Uludağ University School of Medicine, Pediatric Neurology, Bursa, Turkey; +90 224 442 81 43edpapatya@yahoo.comUludağ University School of Medicine, Pediatric Endocrinology, Bursa, Turkey

**Keywords:** Autoimmune oophoritis, myasthenia gravis, thymectomy

## Abstract

Myasthenia gravis (MG) is an autoimmune disorder characterized  by autoantibodies against acetylcholine receptors. MG is generally an isolated disorder but may occur concomitantly with other autoimmune diseases. We describe an eighteen-year-old girl with MG who was admitted to our clinic with secondary amenorrhea and diagnosed as autoimmune oophoritis. Since her myasthenic symptoms did not resolve with anticholinesterase therapy, thymectomy was performed. After thymectomy, her menses have been regular without any hormonal replacement therapy. To our knowledge, this is the first report on a patient with autoimmune ovarian insufficiency and MG in whom  premature ovarian insufficiency resolved after thymectomy, without  hormonal therapy.

**Conflict of interest:**None declared.

## INTRODUCTION

Myasthenia gravis (MG) is an autoimmune disorder characterized by autoantibodies against acetylcholine receptors (1). MG is generally an isolated disorder but a minority of patients may develop MG in conjunction with other autoimmune organ failures such as autoimmune thyroid diseases, rheumatoid arthritis, pernicious anemia and systemic lupus erythematosus (2,3,4,5). Premature ovarian failure (POF) has been very rarely reported in association with MG. We report a case with MG in whom POF has resolved after thymectomy.

## CASE REPORTS

An eighteen-year-old girl with a six-month-history of secondary amenorrhea was admitted to our pediatric endocrinology clinic. Her past medical history was remarkable for MG, presenting with ptosis and generalized muscle weakness developing at the end of the day. She was fifteen years old when she was diagnosed as having autoimmune MG. Her symptoms were partially resolved with pyridostigmine bromide (120 mg/day) and at the eleventh month of this therapy, prednisolone (60 mg/day) was added. After two months of prednisolone treatment, psychiatric symptoms appeared which were attributed to drug-induced bipolar affective disorder and steroid treatment was gradually discontinued. The antipsychotic olanzapine (15 mg/day) was started. Her menstrual cycles had started when she was twelve years old and were regular until thirteen months after the diagnosis of MG and at the third month of olanzapine therapy, when menstrual irregularity had appeared. Her family history was unremarkable. There was no pathologic finding in her physical examination. Her pubertal development was evaluated as Tanner stage 5.

Since neuroleptic drugs can increase serum prolactin levels and cause menstrual irregularity, the patient’s prolactin level was checked and found normal. Her gonadotropin and inhibin B levels were in postmenapausal ranges (follicle stimulating hormone: 56.23 mIU/mL, luteinizing hormone  25.43 mIU/mL, inhibin B<10 pg/mL) and estrogen level was 18 pg/mL. The patient’s hormonal levels are shown chronologically from the beginning of the menstrual irregularity in Table 1. Primary ovarian insufficiency was evident with these hormonal levels. Her suprapubic pelvic ultrasonography revealed a large cystic formation (57x95x112 mm) in her left ovary. For etiological investigation of autoimmune POF (APOF), we checked antiovarian antibodies and the antibodies against the 21-hydroxylase enzyme. They were all negative. Acetylcholine receptor antibodies were found to be positive at the beginning of the myasthenic symptoms. Antithyroid peroxidase and antityroglobulin antibodies were also negative. We could not measure steroidogenic cell antibodies. The patient’s myasthenic symptoms did not resolve with anticholinesterase therapy, and thymic enlargement was detected by thorax magnetic resonance imaging. Since there is a general consensus that patients with generalised MG between puberty and 60 years of age may benefit from thymectomy, this surgical procedure  was performed and pathologic examination revealed follicular thymic hyperplasia. Three months after thymectomy, her regular menses started. Gonadotropin levels decreased to normal ranges, but her left ovarian cyst persisted. Six months after thymectomy, her left ovarian cyst was removed. Pathological diagnosis was benign mucinous cyst. Her menstrual bleeding has been regular since thymectomy, without any hormonal replacement therapy.

**Table 1:**
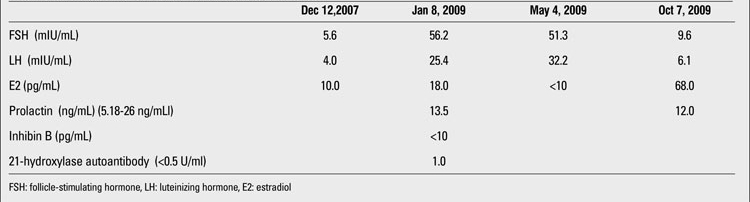
Laboratory evaluation of the patient in a chronological order

## DISCUSSION

POF is characterized by amenorrhea, hypoestrogenism and elevated serum gonadotropin levels in women younger than 40 years (6). Among a total of 266 POF patients, 4% of  patients with spontaneous POF were diagnosed to have autoimmune oophoritis (AO) (7). An autoimmune mechanism was described in some patients with lymphocytic and plasma cell infiltration in developing follicles (8,9,10). The only validated marker to detect AO is the presence of steroidogenic cell autoantibodies (7, 11). 

Coexistence of autoimmune ovarian failure and other autoimmune diseases was reported to be between 10% and 55% (6,12,13).  APOF could be seen as a part of autoimmune 1polyglandular syndrome type 1 (APGST1) (72%) and APGST2 (10%) at the age of 40 years (14). It was also reported that 12-33% of women who presented with APOF had autoimmune thyroid disease as well (13,15,16,17). APOF was also observed in patients with systemic lupus erythematosus  and MG (4,18,19,20). APOF and adrenal autoimmunity coexistence is the  best known but not the most frequent association. Histological confirmation of  APOF was first reported in a patient with autoimmune adrenal insufficiency (8). There was lymphocytic infiltration in the secondary and antral follicles, but primordial follicles were spared (21). Lymphocytic infiltration was most intense in the theca layer of the developing follicles and related with the intense luteinization of the follicles (7,8,9,10). Ovarian biopsy is not recommended for the confirmation of the diagnosis in APOF. In one study (22), highly elevated total inhibin and inhibin B serum levels were reported in patients with APOF compared to patients with idiopathic POF and natural menopause. Increased inhibin B level was thought to be  a result of the initial preservation of granulosa cell functions. As the disease advances, ovaries can become atrophic. In our patient, inhibin B level was in postmenauposal range and estrogen levels were low. We thought that this postmenopausal inhibin B level was the result of an advanced stage of the ovarian destruction in our patient. We did not perform ovarian biopsy at the time of diagnosis. However, six months after thymectomy, a benign mucinous ovarian cyst was removed. The pathology of the cyst showed no lymphocytic and plasma cell infiltration. At the time of the ovarian operation, our patient had been having regular menstrual bleeding and normal gonadotropin and sex steroid levels for six months. We thought that this was the reason we could not find lymphocytic and plasma cell infiltration representing AO.  However, it has been recently reported that lymphocytic and plasma cell infiltration may be absent and selective mononuclear cell infiltration free of lymphocytes may be observed in AO (23). Antibodies against steroid cell antigens obtained from adrenal tissue substrates are the most validated marker for the diagnosis of APOF that correlates with ovarian inflammation (7). We could only study anti-21-hydroxylase and antiovarian antibodies and they were negative. The predictive value of the commercially available antiovarian antibody test is poor. One study (24) showed that these tests use animal ovarian tissues and have an unacceptably high false-positive rate.

Large ovarian cysts have been reported in several patients with AOF. As a result of follicular dysfunction, impaired negative feedback lead to elevated gonadotropin levels which overstimulate the ovarian tissues and lead to the development of large luteinized ovarian cysts (25,26,27,28,29). Initially, we thought that our patient’s large ovarian cyst had developed with this mechanism. However, we did not observe any regression in this large ovarian cyst after thymectomy although her regular menstruation resumed.  On the other hand, thymectomy has been reported to induce autoimmune ovarian dysgenesis and autoimmune dacryoadenitis in mice. However, the significance of this finding in humans especially in the setting of MG is not clear (30).

Coexistence with another autoimmune disease is not a criterion for the diagnosis of APOF. However, our patient had primary ovarian failure and her ovarian insufficiency resolved after thymectomy. For this reason, we thought she had AO. 

There is no controlled research about the treatment of AOF, but steroid therapy 20-40 mg/day and cyclic estrogen-progesterone replacement therapy have been reported to be successful in several case reports and case series in adults (31). We did not use steroid therapy, because our patient had developed steroid-related bipolar affective disorder during the therapy for MG. In 1969, Lundberg and Person   reported a 25-year-old MG female patient with POF and her menstrual cycles became regular eight weeks after thymectomy (5). The authors thought that her ovarian failure had an autoimmune etiology.  In 1993, a 27-year-old patient with POF and MG was reported to have had thymectomy one year after the initial diagnosis during which she received   pyridostigmine and hormone replacement therapy. Spontaneous pregnancy occurred three years after thymectomy (32). 

Our patient was not on hormonal replacement before and after thymectomy. Her regular menses started three months after thymectomy. To our knowledge, this is the first report on a patient with autoimmune ovarian insufficiency and MG in whom premature ovarian insufficiency resolved after thymectomy without hormonal therapy. We therefore suggest that treatment of MG including thymectomy if indicated may lead to resolution of the ovarian failure and hormonal therapy may not be necessary. If hormonal therapy had been 

introduced before MG was successfully treated; a trial of discontuniation of cyclic hormone replacement may reveal ovarian recovery.
